# Supplementary material for: Complexome profiling of the Chlamydomonas psb28 mutant reveals TEF5 as an early PSII assembly factor
Source: Plant Cell. 2025 Mar 17;37(6):koaf055. doi: 10.1093/plcell/koaf055 (PMC12164590; doi:10.1093/plcell/koaf055)
Supplement: koaf055_Supplementary_Data [file koaf055_supplementary_data.zip › psb28_interactive.zip!/plots/intensity/qualityPlots/peptides/atpA_Cre-1.g2717041.t1.p1.html]

### Cre-1.g2717041.t1.p1

Trivial name: atpA  
SeqCoverage [median]: 58.30  
Mass: 54.75 kDa  
  
 

| Sequence | MissedCleavage | Mass [Da] | wtIntensity | muIntensity | Score |
| --- | --- | --- | --- | --- | --- |
| AIESPAPGIVAR | 0 | 1179.66 | 2584662.38 | 2184571.77 | 282.51 |
| AIESPAPGIVARR | 1 | 1335.76 | 0.00 | 26468.74 | 93.84 |
| AMRTPEELSNLIK | 1 | 1500.80 | 40550.21 | 66357.93 | 190.27 |
| AMRTPEELSNLIKDLIEQYTPEVK | 2 | 2816.46 | 2149.82 | 0.00 | 87.51 |
| ASSVAQVLNTLK | 0 | 1229.70 | 2089695.37 | 2750263.80 | 334.13 |
| ASSVAQVLNTLKER | 1 | 1514.84 | 135131.37 | 516771.77 | 194.48 |
| AYLSGLR | 0 | 778.43 | 1806664.75 | 974343.55 | 196.46 |
| DLIEQYTPEVK | 0 | 1333.68 | 460819.07 | 395043.70 | 247.51 |
| EAYPGDVFYLHSR | 0 | 1552.73 | 2053289.79 | 3456905.14 | 176.87 |
| EILKQPQSSPLSVEEQVASLYAGTNGYLDKLEVSQVR | 2 | 4075.11 | 13360.91 | 0.00 | 101.78 |
| EMSLLLR | 0 | 860.48 | 1543039.93 | 1441421.80 | 158.38 |
| IAEIPVGEAYLGR | 0 | 1386.75 | 2973484.45 | 3271714.64 | 234.67 |
| IYGLEK | 0 | 721.40 | 1609122.27 | 946529.00 | 133.99 |
| LEVSQVR | 0 | 829.47 | 114459.61 | 127505.15 | 181.49 |
| LREILK | 1 | 770.50 | 0.00 | 341477.72 | 143.02 |
| MVDFGIVFQVGDGIAR | 0 | 1722.88 | 28249.55 | 0.00 | 176.51 |
| PTLTIYDDLSK | 0 | 1264.66 | 42544.57 | 68326.36 | 295.34 |
| PVDGKGAVQTK | 1 | 1098.60 | 416.86 | 0.00 | 38.40 |
| QAINEYLEEFK | 0 | 1382.67 | 447845.56 | 624342.68 | 295.31 |
| QAINEYLEEFKSQAK | 1 | 1796.89 | 877974.69 | 1089419.39 | 257.52 |
| QAQAYR | 0 | 735.37 | 501073.15 | 270327.24 | 115.83 |
| QAQAYREMSLLLR | 1 | 1577.83 | 148477.48 | 213423.55 | 128.54 |
| QPQSSPLSVEEQVASLYAGTNGYLDK | 0 | 2780.35 | 1514.96 | 0.00 | 81.34 |
| QPQSSPLSVEEQVASLYAGTNGYLDKLEVSQVR | 1 | 3591.81 | 82975.49 | 0.00 | 95.11 |
| QTGKTAIAVDTILNQK | 1 | 1699.95 | 3281.24 | 3378.37 | 68.26 |
| RPPGREAYPGDVFYLHSR | 2 | 2116.06 | 0.00 | 34113.84 | 72.43 |
| RSVYEPLATGLVAVDAMIPVGR | 1 | 2313.25 | 157846.29 | 214411.72 | 212.39 |
| STLTFTPEAEGLVK | 0 | 1491.78 | 2451012.07 | 3859870.84 | 263.11 |
| SVYEPLATGLVAVDAMIPVGR | 0 | 2157.15 | 232022.05 | 272948.16 | 203.85 |
| SVYEPLATGLVAVDAMIPVGRGQR | 1 | 2498.33 | 5492.83 | 0.00 | 69.76 |
| SYLANSYPK | 0 | 1041.51 | 1195477.99 | 1079570.96 | 255.95 |
| SYLANSYPKYGEILR | 1 | 1772.91 | 830.11 | 0.00 | 39.03 |
| TAIAVDTILNQK | 0 | 1285.72 | 2287994.85 | 2916597.27 | 358.56 |
| TAIAVDTILNQKGK | 1 | 1470.84 | 14401.92 | 27933.45 | 162.22 |
| TPEELSNLIK | 0 | 1142.62 | 648186.92 | 549102.12 | 247.75 |
| TPEELSNLIKDLIEQYTPEVK | 1 | 2458.28 | 65844.52 | 10197.47 | 284.34 |
| VGSAAQPK | 0 | 756.41 | 1220229.65 | 1020576.63 | 172.03 |
| VGSAAQPKAMK | 1 | 1086.59 | 503.15 | 0.00 | 73.89 |
| VVDGLAR | 0 | 728.42 | 142717.64 | 89279.80 | 145.88 |
| VVDGLARPVDGK | 1 | 1224.68 | 3808563.76 | 2795353.03 | 170.68 |
| VVDGLARPVDGKGAVQTK | 2 | 1809.01 | 9762.73 | 43582.32 | 58.98 |
| YGEILR | 0 | 749.41 | 1681672.54 | 1289871.01 | 148.37 |
|  |  |  | 31483342.5 | 32972000.9 |  |
